# Serum biomarkers remain stable after transitioning from intravenous to subcutaneous natalizumab in multiple sclerosis

**DOI:** 10.1007/s00415-026-13949-0

**Published:** 2026-06-22

**Authors:** Albert Muñoz-Vendrell, Pablo Arroyo-Pereiro, Isabel León, Laura Bau, Elisabet Matas, Lucia Romero-Pinel, Sergio Roca-Pereira, Antonio Martínez-Yélamos, Sergio Martínez-Yélamos

**Affiliations:** 1 Multiple Sclerosis Unit, Neurology Department, Hospital Universitari de Bellvitge, Carrer de la Feixa Llarga S/N, 08907 L’Hospitalet de Llobregat, Barcelona Spain; 2 Neurologic Diseases and Neurogenetics Group, Neuroscience Program, Institut d’Investigació Biomèdica de Bellvitge (IDIBELL), L’Hospitalet de Llobregat, Barcelona Spain; 3 Network Centre of Biomedical Research of Neurodegenerative Diseases (CIBERNED), Institute of Health Carlos III, 08907 L’Hospitalet de Llobregat, Barcelona Spain; 4 Departament de Ciències Clíniques, Facultat de Medicina, Universitat de Barcelona, Barcelona, Spain

**Keywords:** Multiple sclerosis, Neurofilament light chain, Glial fi brillary acidic protein, Biomarkers, Natalizumab

## Abstract

**Background:**

Subcutaneous natalizumab offers greater convenience than intravenous administration, but pharmacokinetic differences have raised concerns about potential subclinical disease activity. Serum neurofilament light chain (sNfL) and glial fibrillary acidic protein (GFAP) are sensitive biomarkers of neuroaxonal damage and astroglial activation.

**Methods:**

In this prospective, single-center cohort study, consecutive patients with relapsing–remitting multiple sclerosis who transitioned from intravenous to subcutaneous natalizumab were followed for 12 months. Serum sNfL and GFAP were measured at baseline (prior to switch), and at 6 and 12 months using SIMOA technology. Additional clinical outcomes included annualized relapse rate (ARR), EDSS, and MRI activity.

**Results:**

23 patients were included (mean age 43.7 years; 91% female). Median disease duration was 14 years (IQR 8.1–22), median time on natalizumab was 5 years (IQR 3.3–7.9), baseline 2-year ARR was 0.09 ± 0.2, and median EDSS was 2.0 (IQR 2.0–3.5). Median sNfL *Z*-scores were 0.2 (IQR − 0.3–0.6) at baseline, 0.1 (IQR − 0.4–1.0) at 6 months, and 0.5 (IQR − 0.4–1.1) at 12 months, with no significant change over time (*p* = 0.401). GFAP levels were similarly stable (87.7, 86.8, and 90.2 pg/mL; *p* = 0.957). ARR remained low and unchanged (0.09 pre- and post-switch; *p* = 1.0), with no radiological activity observed. EDSS remained stable over follow-up.

**Conclusions:**

In this small real-world cohort, switching from intravenous to subcutaneous natalizumab was associated with stable sNfL and GFAP levels over 12 months, alongside stable conventional clinical and MRI outcomes. These findings provide supplementary biomarker evidence broadly consistent with existing clinical trial and real-world data, but should be interpreted cautiously given the small sample size.

## Introduction

Natalizumab represented a major therapeutic advancement in multiple sclerosis (MS) nearly 20 years ago. As a humanized monoclonal antibody targeting the α4-integrins on leukocytes, it prevents leukocyte migration across the blood–brain barrier and reduces central nervous system inflammation [1]. Its therapeutic efficacy was established in the pivotal AFFIRM trial, with robust real-world evidence further supporting effectiveness and safety [2, 3].

Despite its efficacy, intravenous administration imposes practical burdens, including approximately 2.5 h per monthly infusion [4]. This led to a subcutaneous formulation, authorized by the European Commission in 2021 [European Medicines Agency. Tysabri (natalizumab) EPAR]. Clinical investigations, notably the phase 2 REFINE study, demonstrated non-inferiority to intravenous dosing on MRI activity and relapse rate, alongside greater convenience and strong patient preference for subcutaneous administration [5, 6].

Traditional response measures may miss subclinical disease activity. Serum neurofilament light chain (sNfL) has emerged as a leading blood-based biomarker capturing such activity and informing prognosis [7]. Glial fibrillary acidic protein (GFAP) provides complementary information related to astrocytic pathology, which appears more strongly associated with disease progression [8].

In clinical practice, many patients have transitioned from intravenous to subcutaneous natalizumab for greater convenience, a preference clearly demonstrated in patient surveys [9]. However, this transition can lead to decreased drug levels in blood [10]. Although pharmacokinetic differences have not translated into clinically evident differences in efficacy in clinical trials, their implications for subclinical disease activity remain unknown. To date, no study has specifically assessed subclinical disease activity using sNfL or GFAP in patients transitioning from intravenous to subcutaneous natalizumab.

This study aims to assess subclinical disease activity using sNfL and GFAP in patients transitioning from intravenous to subcutaneous natalizumab.

## Methods

### Study design and patients

This observational, prospective cohort study included consecutive patients diagnosed with relapsing–remitting multiple sclerosis (RRMS) who switched from intravenous to subcutaneous natalizumab between April and October 2022 at a tertiary MS center, with at least 12 months of follow-up. Patients were excluded if blood samples were unavailable or if any concomitant neurodegenerative disease was present.

Patients initially received intravenous natalizumab (300 mg) every 4 weeks and were directly transitioned to subcutaneous natalizumab (300 mg; two injections of 150 mg each) at the time of their next scheduled dose. All treatments were administered in the day-infusion unit by nursing staff, with vital signs assessed before and after administration.

Clinical and imaging data were prospectively collected using the European Database for Multiple Sclerosis (EDMUS) software [11]. Clinical data were obtained by a qualified neurologist during scheduled 6-month follow-up visits at our specialized MS unit and additional visits when relapse was suspected. A relapse was defined as new neurological symptoms or signs lasting more than 24 h in the absence of intercurrent illness, regardless of changes in EDSS score or MRI findings. Magnetic resonance imaging (MRI) was performed annually per protocol, and all scans were evaluated by a qualified neuroradiologist.

### Serum collection, processing, and laboratory analysis

Blood samples were prospectively collected one month before the first subcutaneous dose and after 6 and 12 months of subcutaneous treatment. Serum was centrifuged at 3000 rpm for 15 min at room temperature, aliquoted into 500 μL volumes, and stored at − 80 °C until analysis. Samples were stored and provided by Biobanc HUB-ICO-IDIBELL, funded by Instituto de Salud Carlos III (PT20/00171) and Xarxa de Bancs de Tumors de Catalunya, sponsored by Pla Director d’Oncologia de Catalunya (XBTC).

Serum levels of neurofilament light chain (sNfL) and glial fibrillary acidic protein (GFAP) were measured at baseline and after 6 and 12 months using the NeuroPlex-4B kit and single-molecule array (SIMOA) technology (Quanterix, Billerica, MA, USA). All samples were analyzed in a single run, with biomarker values within the manufacturer’s specified detection range.

For sNfL, age and body mass index (BMI) corrected standard deviation (SD) scores relative to healthy control reference values were calculated using a web-based application derived from Benkert et al. [12].

### Variables and outcome measures

Variables collected included sex, baseline height and weight, age at MS onset, age at initiation of the first disease-modifying treatment (DMT), prior DMTs, age at transition from intravenous to subcutaneous natalizumab, annualized relapse rate (ARR) 2 years before baseline and relapses during follow-up, relapse-free time (RFT) at baseline, radiological activity before and after transition, baseline and follow-up EDSS scores, and sNfL and GFAP levels at baseline, 6, and 12 months.

The primary endpoint was the longitudinal change in age- and BMI-adjusted sNfL *Z*-scores and GFAP levels following the transition from intravenous to subcutaneous natalizumab at baseline, 6 months, and 12 months. Secondary endpoints included clinical relapse occurrence, radiological activity, and EDSS progression during the 12-month follow-up period.

### Statistical analysis

Data distribution was assessed using Shapiro–Wilk test. Continuous variables are presented as mean ± standard deviation (SD) or median and interquartile range (IQR), depending on distribution. Categorical variables are described as frequencies. Longitudinal changes in sNfL and GFAP across the three time points were assessed using Friedman test. Given the absence of a significant global effect, no formal post hoc pairwise comparisons were required; however, exploratory pairwise comparisons using Wilcoxon signed-rank test were also performed and yielded consistent non-significant results. Analyses were performed using complete-case data as no missing biomarker values were observed at the three time points. ARR and EDSS change across 1-year follow-up was assessed using Wilcoxon test. Violin plots were generated to visualize biomarker distributions across time points. A significance level of 5% (95% confidence intervals) was set for all statistical tests. Analyses were performed using SPSS version 20.0 and Stata 19.0. Given the exploratory nature of this study and the limited availability of eligible patients, no formal sample size calculation was performed.

## Results

A total of 23 patients were included, with a mean age of 43.7 ± 10.4 years; 91% were female. The median disease duration since MS onset was 14 years (IQR 8.1–22), median time since initiation of the first DMT was 10.2 years (IQR 5–13.3), and median duration of natalizumab treatment was 5 years (IQR 3.3–7.9). Baseline 2-year ARR was 0.09 ± 0.2, median RFT was 4.0 years (IQR 2.2–6.9), and median EDSS was 2.0 (IQR 2.0–3.5). The median number of prior DMTs before natalizumab was 1 (IQR 0–1).

**Fig. 1:**
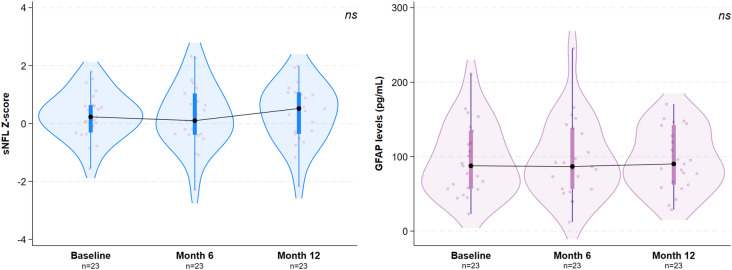
Evolution of serum biomarkers (sNfL *Z*-scores and GFAP) following the transition from intravenous to subcutaneous natalizumab at baseline, 6, and 12 months. *sNFL* serum neurofilament light chain, *GFAP* glial fibrillary acidic protein, *ns* not statistically significant

Median sNfL levels were 7.7 pg/mL (IQR 6.6–9.9) at baseline, 8.6 pg/mL (IQR 6.6–10.0) at 6 months, and 9.0 pg/mL (IQR 6.8–10.5) at 12 months, with no statistically significant change observed (*p* = 0.438). Repeated analysis using *Z*-score normalization confirmed these findings: median sNfL *Z*-scores were 0.2 (IQR − 0.3–0.6) at baseline, 0.1 (IQR − 0.4–1.0) at 6 months, and 0.5 (IQR − 0.4–1.1) at 12 months (*p* = 0.401). Median GFAP levels were 87.7 pg/mL (IQR 56.9–134.7) at baseline, 86.8 pg/mL (IQR 56.5–138.3) at 6 months, and 90.2 pg/mL (IQR 62.2–142.1) at 12 months, also without significant variation (*p* = 0.957). Violin plots illustrating serum biomarker evolution after the transition are shown in Fig. 1.

Over 12 months, ARR remained stable at 0.09 ± 0.3 (*p* = 1.0). Two patients presented with suspected relapses: the first developed brachial hypoesthesia that resolved spontaneously within 3 days after the first serum sampling and prior to the subcutaneous transition, without radiological activity or EDSS increase, and biomarkers were essentially stable from baseline to month 6 (sNfL 7.3 → 9.1 pg/mL; GFAP 140.0 → 138.3 pg/mL). The second patient developed worsening paraparesis 11 months after the transition, with no radiological activity but an EDSS increase from 3.0 to 4.0; this was considered a possible transition to SPMS, with modest increases in sNfL and GFAP from month 6 to month 12 (7.8 → 9.9 pg/mL and 86.8 → 127.9 pg/mL, respectively). No radiological activity was detected in any patient during the 12-month follow-up. Two additional patients experienced EDSS progression in the absence of relapse or radiological activity (from EDSS 2.0 to 2.5, and from 3.0 to 4.0): in the first, sNfL was unchanged while GFAP increased (11.9 → 11.9 pg/mL and 77.4 → 131.0 pg/mL); in the other, sNfL increased with a small GFAP rise (7.6 → 19.1 pg/mL and 45.1 → 51.0 pg/mL). Overall, the median EDSS remained 2.0 (IQR 2.0–4.0; *p* = 0.102).

## Discussion

In this prospective, observational study, we evaluated subclinical disease activity through serum biomarkers (sNfL and GFAP) in RRMS patients transitioning from intravenous to subcutaneous natalizumab. sNfL and GFAP remained stable over the 12-month follow-up, indicating no significant change in subclinical disease activity following the change in administration route. Clinically, ARR and median EDSS were unchanged, and no new MRI activity was detected. These findings suggest that pharmacokinetic differences associated with subcutaneous dosing do not translate into increased neuroaxonal damage or astroglial activation as reflected by stable sNfL and GFAP levels.

In patients treated with intravenous natalizumab, NfL levels decline markedly after treatment initiation and remain low as inflammatory activity is controlled, with approximately threefold reductions in CSF by 6–12 months [13] and ~ 60–70% decreases in serum over the same period, particularly among patients achieving NEDA-3 [14]. By contrast, GFAP shows more heterogeneous behavior: group-level changes are small, modest declines appear in responders [14], and serum GFAP tends to track progression more than acute inflammatory activity [15]; its prognostic value under natalizumab is therefore inconsistent.

Although previous studies have raised concerns regarding potential decreases in drug exposure after switching formulations [10], larger studies have demonstrated similar efficacy between intravenous and subcutaneous natalizumab [5]. Together with these data, our findings indicate that, over 12 months, subclinical inflammatory activity remains stable following transition to the subcutaneous formulation, with parallel stability in clinical outcomes. Our results align with earlier studies reporting non-inferior clinical efficacy and patient preference for the subcutaneous formulation. The REFINE study [5] and subsequent post hoc analysis [16, 17] showed comparable efficacy between intravenous and subcutaneous natalizumab, emphasizing benefits, such as improved convenience and patient satisfaction [6, 18]. Our biomarker data further complement these studies by providing supplementary biological evidence consistent with sustained subclinical disease control.

The isolated instances of relapse and EDSS worsening observed in this study appear consistent with the natural history of RRMS rather than reduced treatment efficacy. Overall, neither mean ARR nor median EDSS changed relative to the pre-transition period. The only relapse after the switch occurred 11 months later, without radiological activity and with only modest sNfL/GFAP increases. Two additional patients showed EDSS worsening with mild biomarker rises in the absence of clear relapse or MRI activity, consistent with the closer association of GFAP with progressive biology [15]. These findings highlight the complexity of clinical evaluation in MS and suggest that occasional symptom fluctuations—and even transition to SPMS—may occur despite stable therapeutic efficacy and independently of ongoing high-efficacy DMT [19].

The strengths of this study include its prospective design, the homogeneity of the patient population and methodology, and the use of validated biomarkers for assessing subclinical disease activity. The main limitation of this study is its very small sample size, which substantially limits statistical power to detect modest biomarker changes or infrequent clinical and radiological events, and restricts the generalizability of our findings. Additionally, the follow-up duration of 12 months, while adequate for initial observations, may be insufficient to detect longer-term trends or delayed treatment effects. In addition, MRI assessment was limited to routine annual clinical MRI evaluating new or enlarging T2 lesions and gadolinium-enhancing lesions. The absence of a comparative control group, particularly patients continuing intravenous natalizumab, is another important limitation. Therefore, we cannot determine whether biomarker stability was specifically related to the transition to subcutaneous natalizumab or reflected the natural course of an already stable cohort with very low pre-switch disease activity. The single-center design further restricts the broader applicability of our findings.

Future research should expand upon these findings through multi-center studies with larger cohorts and longer-term follow-up. Additional biomarkers and advanced imaging measures could enhance our understanding of subclinical changes associated with different treatment modalities. Pharmacokinetic assessments might also help clarify relationships between drug exposure levels, biomarkers, and clinical outcomes.

In conclusion, in this small, clinically stable RRMS cohort, transitioning from intravenous to subcutaneous natalizumab was associated with stable sNfL and GFAP profiles over 12 months, with no evidence of increased subclinical disease activity as assessed by these biomarkers. These findings should be interpreted cautiously given the limited sample size and absence of a control group, but provide supplementary biomarker evidence that is broadly consistent with existing clinical trial and real-world data supporting maintained disease control after transition to subcutaneous natalizumab.

## Data Availability

The datasets used and/or analyzed during this study are available from the corresponding author on reasonable request from any qualified investigator.
